# Differentiated muscles are mandatory for gas-filling of the *Drosophila* airway system

**DOI:** 10.1242/bio.013086

**Published:** 2015-11-30

**Authors:** Yiwen Wang, Tina Cruz, Uwe Irion, Bernard Moussian

**Affiliations:** 1 Animal Genetics, Interfaculty Institute for Cell Biology, University of Tübingen, Auf der Morgenstelle 15, Tübingen 72076, Germany; 2 Department of Genetics, Max-Planck Institute for Developmental Biology, Spemannstr. 35, Tübingen 72076, Germany; 3 Institute of Biology Valrose, University of Nice, Parc Valrose, Nice 06108, France; 4 Applied Zoology, Department of Biology, Technische Universität Dresden, Zellescher Weg 20b, Dresden D-01217, Germany

**Keywords:** *Drosophila*, Differentiation, Embryo, Gas-filling, Organ, Trachea

## Abstract

At the end of development, organs acquire functionality, thereby ensuring autonomy of an organism when it separates from its mother or a protective egg. In insects, respiratory competence starts when the tracheal system fills with gas just before hatching of the juvenile animal. Cellular and molecular mechanisms of this process are not fully understood. Analyses of the phenotype of *Drosophila* embryos with malformed muscles revealed that they fail to gas-fill their tracheal system. Indeed, we show that major regulators of muscle formation like Lame duck and Blown fuse are important, while factors involved in the development of subsets of muscles including cardiac and visceral muscles are dispensable for this process, suggesting that somatic muscles (or parts of them) are essential to enable tracheal terminal differentiation. Based on our phenotypic data, we assume that somatic muscle defect severity correlates with the penetrance of the gas-filling phenotype. This argues that a limiting molecular or mechanical muscle-borne signal tunes tracheal differentiation. We think that in analogy to the function of smooth muscles in vertebrate lungs, a balance of physical forces between muscles and the elasticity of tracheal walls may be decisive for tracheal terminal differentiation in *Drosophila*.

## Abstract (precis)

**Summary:** During embryogenesis in *Drosophila melanogaster*, without involving the nervous system, somatic muscles control terminal differentiation of the airway system by stimulating gas-filling before hatching.

## INTRODUCTION

Birth occurs when organs of an organism are differentiated to a point allowing survival outside the protective body of the mother or the egg. Organ differentiation during development is mostly reported to follow an intrinsic genetic, molecular and cellular program. Hormones emanating from the brain have been shown to orchestrate morphogenesis, in turn also phasing differentiation (Chavoshi et al., 2010; Ruaud et al., 2010). In this work we have been following the question whether there is any communication between the organs of an organism during terminal differentiation, i.e. do organs coordinate their differentiation?

At the end of insect embryogenesis, the larva fills its respiratory system with gas, moves coordinately to tear apart the eggshell and eventually hatches. This behaviour implies that the brain, the tracheal system, the musculature and the exoskeleton are terminally differentiated i.e. ready to allow or support hatching. We have observed that *Drosophila melanogaster* larvae homozygous mutant for *muscleblind* (*mbl*) coding for a splicing factor (Artero et al., 1998; Irion, 2012), that amongst others is needed for muscle formation in vertebrates and invertebrates, fail to gas-fill their tracheal network. We therefore asked whether muscles might influence tracheal terminal differentiation.

Tracheal development starts soon after pattern formation when paired clusters of cells in the ectoderm of each body segment are specified to invaginate and form tubes that eventually fuse to give rise to a reticulate airway system (Samakovlis et al., 1996). Cell movement during invagination and fusion has been shown to depend on correct distribution of mesodermal cells that will form the musculature (Franch-Marro and Casanova, 2000). Later in development, tracheal morphogenesis seems to follow an intrinsic program of tube length and diameter determination. A luminal chitin rod plays a central role in these processes (Tonning et al., 2005). Indeed, mutations in chitin synthesis and organizing factors cause aberrant tube length and diameter (Tonning et al., 2005, 2006; Luschnig et al., 2006; Moussian et al., 2006b, 2015). Once tube size is established a fortifying cuticle is deposited at the apical side of tracheal cells (Moussian et al., 2006a), while the luminal chitinous matrix is removed in pulses of endocytosis and finally replaced by gas by an unknown mechanism (Tsarouhas et al., 2007). In parallel to tracheal development, the mesoderm ultimately gives rise to the musculature that is subdivided into three types of tissues. Somatic muscles anchored at epidermal muscle attachment sites allow locomotion, visceral muscles allow food transport during digestion and heart muscles allow haemolymph circulation.

Our genetic data indicate that at the end of embryogenesis somatic muscles are needed for tracheal terminal differentiation. Despite some efforts, we could not define the subtype of muscles responsible for this function. We propose that a certain mass of intact muscle is necessary for gas-filling. Two alternative mechanisms are plausible. First, muscles may produce a molecular signal that accumulates in time to tune tracheal differentiation after a threshold is reached. Another possibility is that physical forces like tension and contraction may in concert with tracheal wall elasticity mechanically stimulate gas bubble formation in tracheal tubes. Either of the mechanisms would, once unravelled, contribute to our understanding of the old question of insect tracheal gas-filling formulated 150 years ago (Weismann, 1863).

## RESULTS

### Mbl is needed for tracheal gas-filling

**Fig. 1.**
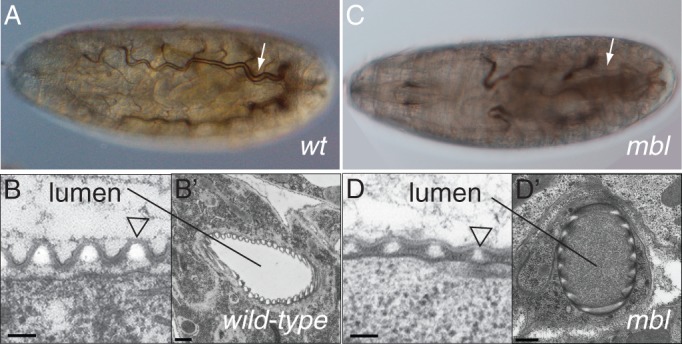
**Mutations in *mbl* prevent tracheal gas-filling.** (A) Wild-type ready to hatch embryos fill their tracheal system (arrow) with gas. When filled with gas the tracheal system becomes visible (dark) due to different refraction indices of gas and liquid. See this effect also in Movie 1. (B,B′) The tracheal cuticle (triangle) lines the surface of tracheal epithelial cells and stabilises the tube. (C) Mutations in the *mbl* gene impair tracheal gas-filling (arrow indicates tracheal system). (D,D′) The tracheal tubes of *mbl* mutant animals are nevertheless present, have a normal cuticle (triangle) but are occasionally filled with material. A,C: Nomarski optics; B,D: transmission electron microscopy. Scale bars: 100 µm in B and D; 250 µm in B′ and D′.

**Fig. 2.**
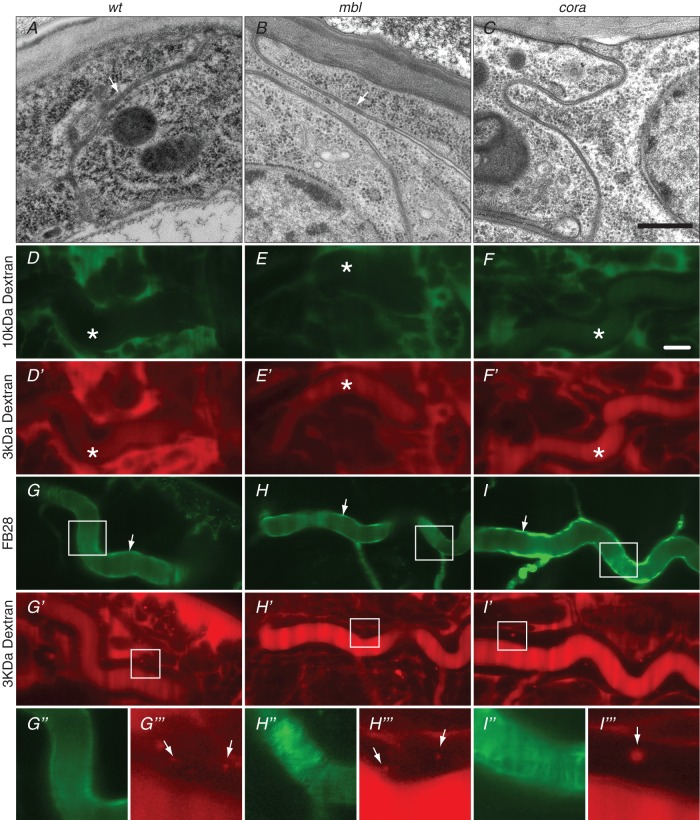
**Mbl is not required for epithelial barrier differentiation.** (A-C) The paracellular barrier of wild-type (A) epithelial cells is constituted by lateral septate junction (white arrows). Septate junctions are present in *mbl* mutant embryos (B), while in *cora* mutant animals they are missing (C). (D-F′) 3 kDa dextran conjugated with rhodamine injected into living wild-type (D) or *mbl* mutant (E) embryos at early stage 17 before tracheal gas-filling penetrates the lumen of tracheae (*) whereas 10 kDa dextran conjugated with FITC does not (D′,E′). In *cora* mutant embryos, both dextrans do leak into the tracheae (F,F′). (G-I‴) Formation of the tracheal cuticle (arrows) is visualised in live wild-type (G,G″), *mbl* (H,H″) and *cora* (I,I″) animals by the detection of chitin through injected FB28. Injection of 3 kDa rhodamine-dextran reveals the presence of endocytotic vesicles (arrows) containing luminal material in these embryos (G′,G‴,H′ H‴,I′ I‴). A-C: electron microscopy; D-I: confocal microscopy. Scale bars: 500 μm in A-C; 10 μm in D-I′.

When we were analysing the phenotype of stage 17 ready-to-hatch embryos homozygous mutant for the *mbl* gene in order to learn more about the role of this gene on embryo differentiation, we noticed that these animals failed to fill their tracheal tubes with gas (Fig. 1). In a simplified scenario, five composite cellular mechanisms have been reported to be deployed in tracheal cells before gas-filling: degradation of the luminal chitin rod, endocytosis, establishment of a paracellular barrier, cuticle formation and greasing of the lumen surface (Forster and Woods, 2013). To test whether these mechanisms are aberrant in *mbl* mutant embryos, we performed injection assays with fluorescence dyes in wild-type and *mbl* mutant embryos. Distribution of dyes was monitored by time-lapse confocal microscopy. Injection of 3 and 10 kDa Dextran conjugated with FITC and Rhodamine, respectively, revealed that both endocytosis and the paracellular barrier are normal in *mbl* mutant embryo (Fig. 2). Injection of fluorescence brightener 28 (FB28) that is used to detect chitin (Moussian et al., 2005; Wang et al., 2015) showed that the luminal chitin is formed and degraded in the lumen in all animals studied. In later embryos, FB28 binds to the apical site of tracheal cells lining the differentiating cuticle. In electron-micrographs of wild-type late stage 17 embryos the surface of the tracheal lumen is lined by the outermost cuticle layer, the envelope that contains lipids. The surface of the tracheal cuticle in *mbl* mutant embryos is unchanged. Taken together, gas-filling is defective in *mbl* mutant embryos although endocytosis, luminal chitin degradation, paracellular barrier, cuticle formation and surface lipid deposition are normal.

### Somatic muscles are required for tracheal gas-filling

**Fig. 3.**
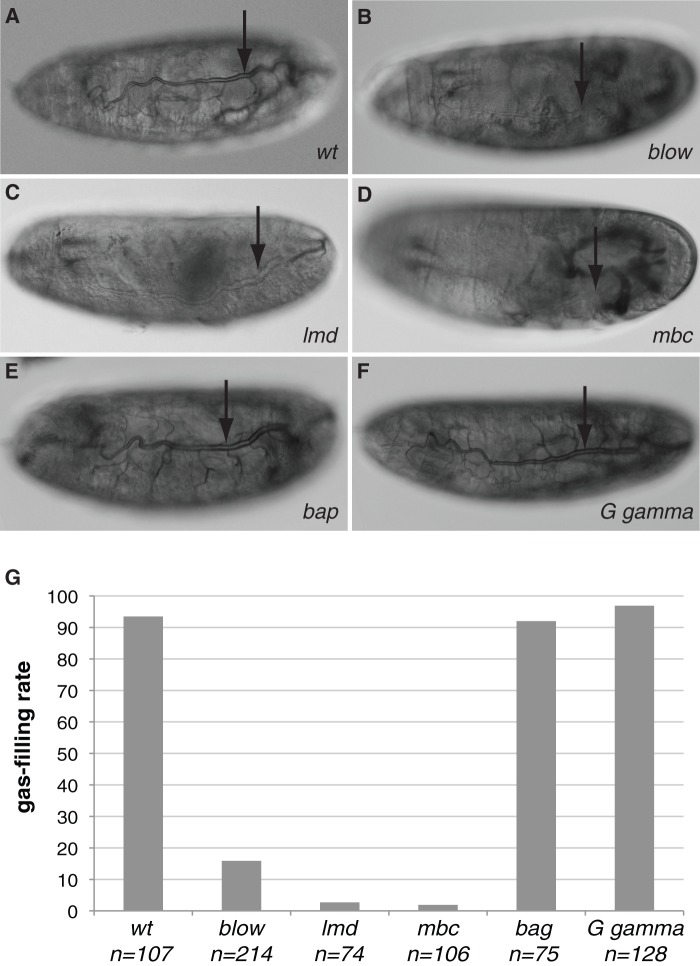
**Mutations in genes required for muscle formation affect tracheal gas-filling.** (A-F) At the end of embryogenesis, the tracheal system (arrow) fills with gas (A). Late embryos mutant for *blow* (B), *lmd* (C) or *mbc* (D) fail to gas-fill their tracheae. Mutations in *bap* (E) or *Gγ* (F), by contrast, do not have a negative effect on tracheal gas-filling. A-F: Nomarski optics. (G) The penetrance of the tracheal gas-filling phenotype in muscle mutants depends on the strength of the mutation. The percentage of embryos with gas-filled tracheae is given. Gas-filling penetrance in wild-type larvae and *bag* and *Gγ* mutant larvae is comparable. In *blow* mutant larvae, the phenotype is well penetrant but milder than in *lmd* and *mbc* mutant larvae.

**Fig. 4.**
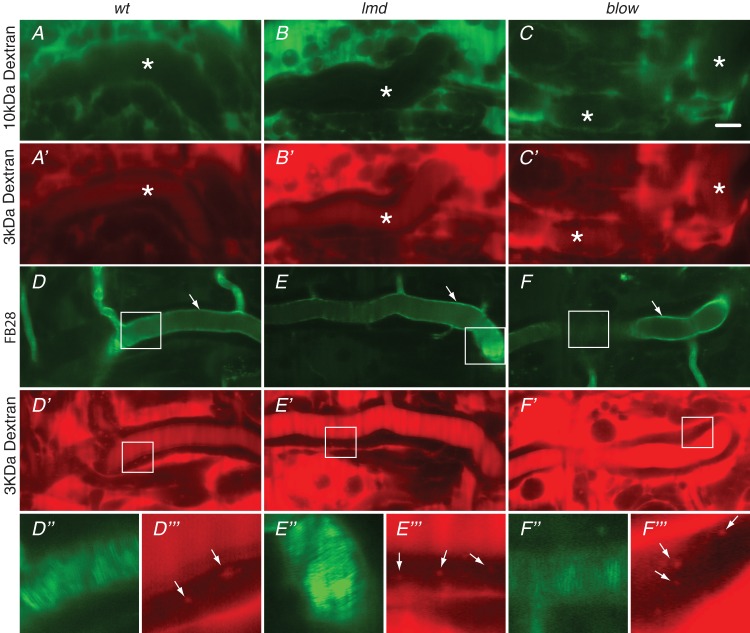
**Mutations in *lmd* and *blow* do not affect tracheal differentiation.** (A-C′) 3 kDa rhodamine-dextran injected into living wild-type (A), *lmd* (B) or *blow* (C) mutant early stage embryos diffuses into the lumen of the tracheal system, whereas injected 10 kDa FITC-dextran is excluded form their lumina (A′-C′). Asterisks indicate the tracheal lumen. (D-F‴) Removal of the luminal chitin rod and cuticular (arrows in D-F) chitin deposition (FB28) and lumen clearance are normal in these embryos (D,D′,E,E′,F,F′). Higher magnifications of the boxed regions in D-F and D′-F′ are shown in D″-F″ and D‴-F‴, respectively. The taenidial folds of the tracheal cuticle are well visible in D″-F″. Arrows in D‴-F‴ point to endocytosed Rhodamine-dextran particles. All images: Confocal microscopy. Scale bar: 10 μm.

Amongst others, Mbl is needed for muscle development opening the possibility that muscles are required for tracheal gas-filling. To test this hypothesis, we studied gas-filling in various embryos homozygous mutant for genes specifically essential for muscle development. Mutations in *myoblast city* (*mbc*), *lame duck* (*lmd*) and *blown fuse* (*blow*), regulating somatic and visceral (Rushton et al., 1995; Doberstein et al., 1997; Duan et al., 2001; Popichenko et al., 2013) muscle development, also affect gas-filling (Fig. 3). The morphology of their tracheae is, however, normal (Fig. S1). Moreover, as in *mbl* mutant embryos, endocytosis, luminal chitin degradation, formation of the paracellular barrier and the tracheal cuticle are unaffected in *lmd* and *blow* mutant animals (Fig. 4).

Mutations in *lmd* that cause a penetrant gas-filling phenotype may have a previously overlooked direct effect on tracheal terminal differentiation. In other words, expression of *lmd* in tracheal cells themselves – in fact, *lmd* expression has not been tested beyond stage 15 (Ruiz-Gomez et al., 2002) – may be necessary for tracheal gas-filling. To challenge this hypothesis, we expressed a wild-type version of the *lmd* gene in tracheal cells of *lmd* mutant embryos using the UAS/Gal4 expression system (Table 1). Of note, expression of wild-type *lmd* under the control of the mesoderm specific Gal4 driver *twist*-Gal4 is able to normalise gas-filling in *lmd* mutant embryos. By contrast, tracheal expression of *lmd* through the tracheal specific Gal4 driver *btl*-Gal4 is unable to restore gas-filling in *lmd* mutant animals. Thus, Lmd does not seem to act in tracheal cells.

**Table 1.**
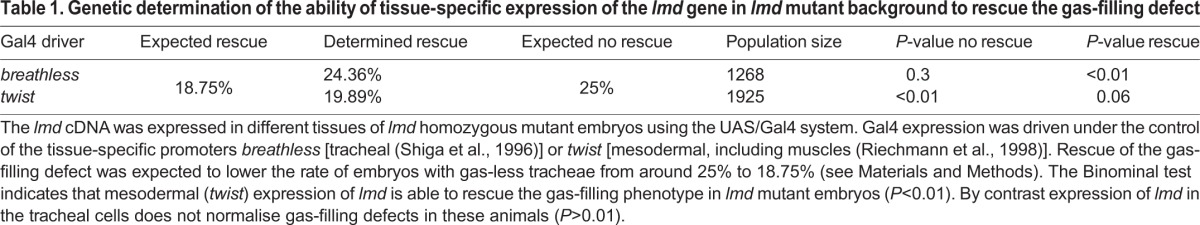
**Genetic determination of the ability of tissue-specific expression of the *lmd* gene in *lmd* mutant background to rescue the gas-filling defect**

For a more detailed analysis we studied the gas-filling ability of mutations affecting only a subset of muscles. Mutations in *kon-tiki* affect the attachment of four ventral-longitudinal muscles (VL1-4) in each segment of the animal to their respective muscle attachment sites (apodemes) (Schnorrer et al., 2007). The tracheae of *kon-tiki* mutant embryos do fill with gas indicating that this gene is not needed for this process (Fig. S2). Specification of some visceral and the alary muscles depends on the function of *optomotor-blind-related-gene-1* (*org-1*) (Schaub et al., 2015). Embryos homozygous for *org-1* mutations do not display any problem with tracheal gas-filling (Fig. S2). A group of dorsal muscles (DO4 and DA3) are missing in *nautilus* (*nau*) mutant embryos (Abmayr and Keller, 1998). Despite this defect, *nau* mutant embryos are able to gas-fill their tracheal system correctly (data not shown).

Mutations in *bagpipe* (*bap*), a gene driving the development of visceral muscles (Azpiazu and Frasch, 1993), do not interfere with gas-filling (Fig. 3). Likewise, mutations in the G-protein γ, which is needed for proper heart muscle morphogenesis (Yi et al., 2006, 2008), do not have any effect on tracheal gas-filling (Fig. 3). This finding is consistent with the observation that *lmd* and *blow* mutant embryos have normal hearts (data not shown). Together, these genetic data indicate that somatic but not visceral or cardiac muscles influence tracheal gas-filling.

### Somatic muscles and tracheae are in physical contact

The tracheal system supplies muscles with air. To understand the extent of physical contact between these organs, we studied the architecture of the tracheal system by Nomarski optics in larvae that express a GFP-tagged version of myosin heavy chain (Mhc-GFP) highlighting muscles by fluorescence microscopy (Fig. S3). The main tubes of the airway system, the dorsal trunks, which are the predominant site of gas-filling, do not contact muscles. By contrast, especially primary and secondary branches distinctly contact muscle fibers. To learn more about this physical contact, we studied the ultrastructure of these organs in stage 17 embryos by transmission electron microscopy (TEM). Primary branches are indeed closely associated with muscles (Fig. S3). These contacts are present in *lmd* and *blow* mutant embryos suggesting that contact alone is not sufficient for functional relationship between these two organs.

Interestingly, two longitudinal muscles enter the posterior spiracles thereby contacting the Filzkörper, which is the ending of the dorsal trunk (Fig. S3). Notionally, this feature may be important for tracheal gas-filling. However, in embryos missing the Filzkörper and by consequence the respective muscle insertion gas-filling is normal (Fig. S3). Thus, proper contact of muscles with the Filzkörper does not play a part in tracheal differentiation. This finding also underlines the observation that gas-filling in the embryo does not imply inflow of atmospheric air through the Filzkörper.

### Metabolic processes in muscles are not needed for tracheal gas-filling

**Fig. 5.**
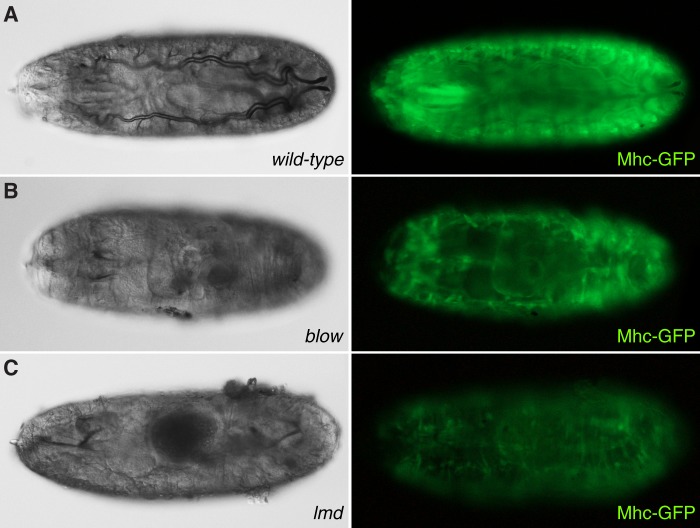
**Mhc-GFP expression is lower in *lmd* than in *blow* larvae.** Expression of GFP under the control of the promoter of the *myosin-heavy chain* gene (*Mhc*) marks muscles or muscle relicts in wild-type (A), *blow* (B) and *lmd* mutant larvae (C). GFP expression is reduced in *blow* and highly reduced in *lmd* mutant larvae compared to wild-type. Left panel: Nomarski optics; right panel: fluorescence microscopy.

Mutations in *blow* cause milder muscle defects than mutations in *lmd* (Fig. 5); in parallel, *blow* mutant animals have a higher tendency for tracheal gas filling than *lmd* mutant animals (Fig. 3). This correlation suggests that gas filling depends on the physiological quality of muscles. Somatic muscles constitute a large organ with a high rate of energy production and consumption. Hence, we speculated that glycolysis and oxidative phosphorylation in these muscles might be a prerequisite for tracheal terminal differentiation. In this scenario, because of reduced muscle mass (Fig. 5), performance of the respiration chain or glycolysis should be alleviated in *mbl*, *blow* and *lmd* homozygous mutant embryos. In genetic experiments, we explored whether downsized metabolic reaction chains may be a reason for gas-filling failure. Importantly, we found that gas-filling in embryos homozygous mutant for *tend* that codes for the cytochrome c oxidase subunit Va (Mandal et al., 2005), a central enzyme of respiration, is normal (Fig. S4). We also suppressed glycolysis specifically in somatic muscles, by expressing a dominant-negative form of the Insulin receptor (InR^DN^) in muscles using the UAS/Gal4 expression system (Fig. S4). As recently published, expression of InR^DN^ in muscles compromises production of pyruvate the product of glycolysis (Tixier et al., 2013). Expressing InR^DN^ in muscles through the muscle-specific Gal4 driver *duf*-Gal4 does not affect tracheal gas-filling. Consistently, global reduction of glycolysis and oxidative phosphorylation in embryos suffering mutations in the transcription regulator estrogen receptor (*Derr*) (Tennessen et al., 2011) does not interfere with tracheal gas-filling (Fig. S4). Thus, in summary, slowed down glycolysis or oxidative phosphorylation do not seem to compromise tracheal gas-filling.

### Muscle contraction may contribute to tracheal gas-filling

**Fig. 6.**
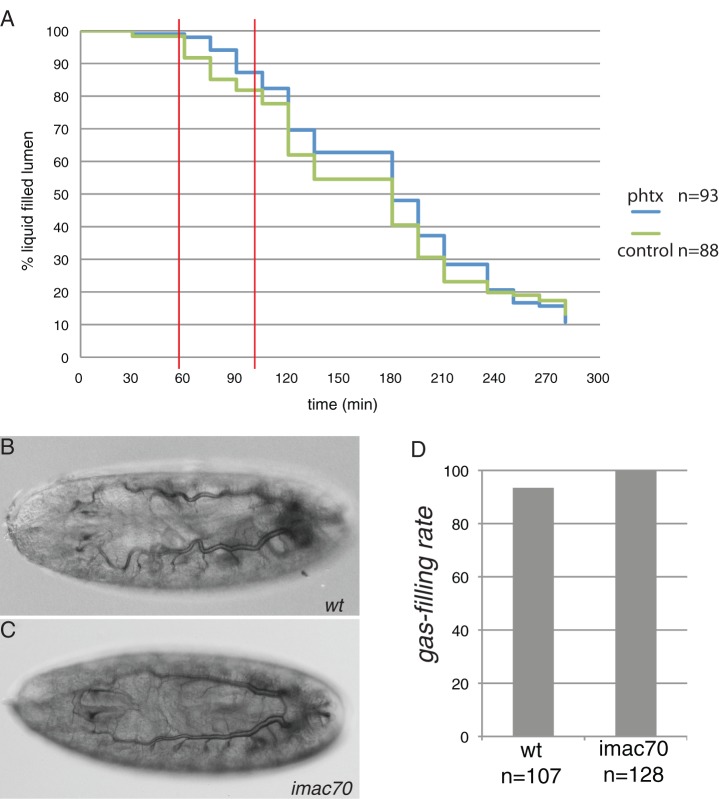
**Muscle enervation is not essential for tracheal gas-filling.** (A) Injection of the glutamate receptor inhibitor philanthotoxin (phtx) into early stage 17 embryos prior to tracheal gas-filling (‘liquid filled lumen’) attenuates tracheal lumen clearance i.e. gas-filling in a time window between one and one and a half hour after injection. According to the Log-rank statistics test, the differences in this period are significant (χ^2^ of 4.1, 6.5 and 4.6 at the time points 60, 75 and 90 min). (B-D) Embryos homozygous for the *imac^170^* mutation fill their tracheae with gas (C), as do wild-type animals (B), with no significant difference detected in gas-filling rate between wild-type and *imac^170^* mutant embryos. B and C, Nomarski optics.

Tracheal gas-filling takes place when embryos start coordinated muscle contraction at the end of embryogenesis (Crisp et al., 2011). It is hence possible that muscle contraction stimulates tracheal terminal differentiation. In agreement with this assumption, *lmd* mutant embryos that do not gas-fill their tracheae are motionless, whereas *blow* mutant embryos that to some degree do gas-fill their tracheal tubes display infrequent contractions (Movies 1, 2 and 3). To scrutinize the relevance of contraction for tracheal differentiation, we injected the spider venom derivative philanthotoxin (Phtx) that inhibits synaptic glutamate receptors at the muscle site (Nakanishi et al., 1990) into mid-stage 17 wild-type embryos. Interestingly, tracheal gas-filling in Phtx injected embryos that do not stop moving was attenuated but not inhibited (Fig. 6). This observation suggests that glutamate receptors are needed for timely lumen clearance and gas-filling.

To challenge these findings, we analysed the ability of embryos with impaired muscle enervation to fill their tracheal system with gas. Embryos homozygous mutant for the *imac* gene that is indispensable for integrity and function of neuro-muscular junctions do not display coordinated muscle peristalsis (Pack-Chung et al., 2007), but fill their tracheal system with gas, as do wild-type embryos (Fig. S5). To follow the behaviour of the organism during gas-filling, we filmed wild-type and *imac* mutant embryos during this time. Wild-type embryos move coordinately at the end of their development, and the tracheal system fills with gas after a contraction bending their anterior region. This contraction is not distinct from previous contractions. Homozygous *imac* mutant embryos hardly move at the end of embryogenesis. However, waves of spontaneous contractions are observed in these embryos. These results support the notion that coordinated muscle contraction as observed in wild-type embryos is not required to stimulate tracheal gas-filling.

## DISCUSSION

Terminal differentiation prepares organisms for life outside the mother or the eggshell. A central question in this issue is whether organs communicate before becoming functional in order to orchestrate differentiation. Early in development, the mesoderm, which is the muscle precursor tissue, assists the patterning of the ectodermal tracheal system in *Drosophila* (Franch-Marro and Casanova, 2000). In the present article, we demonstrate that terminal differentiation of the *Drosophila* tracheal system requires muscle integrity. In turn, functional trachea supply muscles with oxygen required for energy production. Taken together, the dependence of tracheal terminal differentiation on somatic muscle integrity is a keen example for organ-to-organ information flow during embryogenesis.

### Somatic muscles trigger tracheal terminal differentiation

*Drosophila* larvae have three classes of muscles that derive from the mesoderm. Cardiac muscles form the heart, visceral muscles wrap the gut and somatic muscles contact the body wall. The beginning of muscle development is regulated by general mesodermal and muscle genes, while later, additionally, muscle class specific genes drive morphogenesis and differentiation of the respective muscle type (Schnorrer and Dickson, 2004; Richardson et al., 2008). In this work we find that embryos carrying mutations in *lmd* and *blow* coding for regulators of muscle development are unable both to coordinately contract the remnants of their muscles, and to fill their tracheae with gas. Embryos with undeveloped cardiac and visceral muscles, by contrast, have normal tracheae. Hence, our genetic data indicate that somatic muscles are required for tracheal gas-filling, whereas cardiac and visceral muscles are dispensable for this process.

### Gas-filling does not necessitate muscle contraction

Tracheal gas-filling occurs when larvae start contracting their somatic muscles in order to hatch (Crisp et al., 2011). Based on this coincidence and our genetic data that somatic muscles are crucial for tracheal gas-filling, it is conceivable to hypothesize that muscle contraction is needed for tracheal terminal differentiation. Supporting this hypothesis, administration of the neuromuscular junctional glutamate receptor inhibitor Phtx provokes a delay in tracheal gas-filling. By contrast, dysfunctional neuromuscular junctions caused by mutations in the *imac* gene do not interfere with tracheal gas-filling. These animals, however, display waves of spontaneous muscle contractions indicating that coordinated contraction is dispensable for gas-filling. Yet, spontaneous muscle contractions are observed also in *blow* mutant embryos that fail to gas-fill their tracheal system. Thus, taken together, muscle contractions, be it coordinated or spontaneous, are not required for tracheal terminal differentiation.

### Tracheal terminal differentiation correlates with the mass of somatic muscles

The percentage of larvae that are able to fill their tracheae with gas correlates with the degree of Mhc-GFP expression i.e. correct muscle formation. For instance, mutations in *lmd* causing severe defects in large portions of somatic muscles have a significant impact on gas-filling. Weaker muscle malformation as observed in embryos expressing *InR^DN^* (Tixier et al., 2013) is, by contrast, apparently tolerated in this respect. Likewise, mutations affecting a subset of muscles, for example the ventral-longitudinal or the alary muscles, do not interfere with tracheal gas-filling. Certainly, it is not improbable that there is a muscle type specialised in assisting tracheal gas-filling that escaped our analyses. Notwithstanding this argument, the rate of tracheal gas-filling may depend on the mass of intact muscle.

Three alternative effects of muscle mass on tracheal terminal differentiation are plausible.

### Hypothesis 1

Degeneration of muscle tissue may generate a toxic milieu that is unfavourable for tracheal differentiation i.e. gas-filling. Indeed, degeneration of muscles in *mef2* mutant embryos is followed by apoptotic removal of undifferentiated muscle cells (Bour et al., 1995), and these embryos do not gas-fill their tracheae (data not shown). In our ultrastructural analyses of muscles in and acridine orange staining of *lmd*, *blow* and *mbl* mutant embryos we did not find any apoptotic or necrotic muscle cell (Abrams et al., 1993). Thus, intoxication of tracheal cells by derelict muscle cells is probably not the cause of gas-filling failure.

### Hypothesis 2

Tracheal gas-filling may alternatively be induced in response to a threshold amount of a muscle-borne signal. This signal may be a metabolic end-product. The musculature is a large organ that is metabolically very active in the second half of embryogenesis (Tixier et al., 2013). It is hence possible that molecules such as carbon-dioxide produced at the end of glycolysis, the citrate cycle and oxidative phosphorylation accumulate and reach a certain concentration until hatching that is sufficient to trigger gas-filling. Mutations in the *congested-like tracheae* (*colt*) gene that encodes a mitochondrial carrier cause discontinuous tracheal gas content in newly hatched larvae (Hartenstein et al., 1997). It is conceivable that *colt*-deficient mitochondria are impaired in their physiology and by consequence release less metabolic products than normal. In our analyses of larvae suffering mutations (*tend*, *derr* and *InR^DN^*) that affect metabolic reactions, we did, by contrast, not observe gas-filling defects. The very mild *colt* gas-filling phenotype and our observation that mutations in genes active in metabolism do not interfere with gas-filling together suggest that muscle physiology probably does not play a role in tracheal gas-filling.

This interpretation does not object the possibility that muscles emanate another type of signal that dictates tracheal terminal differentiation. In humans, muscles as secretory organs control the function of neighbouring or distant cells. Myokines, for instance, are postulated to bias the physiological state of the organism (Demontis et al., 2013a,b). In an analogous scenario, differentiated muscles in the developing *Drosophila* embryo would produce an endocrine or paracrine signal to influence tracheal behaviour.

### Hypothesis 3

Mechanical information originating from muscles to ensure tracheal integrity is another possible way of organ communication. In theory, gas bubble formation in the insect tracheae has been proposed to be stimulated by pressure reduction on the walls of the tracheal system that in turn induces cavitation (Wigglesworth, 1959; Forster and Woods, 2013). Distortion, bending and straightening of tracheal tubes by contracting and relaxing muscles may actively initiate gas bubbling. We consider this as rather improbable because it is doubtful that the rare and minute movements of *blow* mutant embryos would be effective. Therefore, we favour a view of a more passive influence of the musculature on the tracheal system. This principle is seemingly implemented in the, albeit anatomically different, vertebrate respiratory system. Airway smooth muscles are needed to stabilize bronchial and alveolar structure already during human fetal development (Kim and Vu, 2006; Jesudason, 2009; Prakash, 2013). Erroneous and elevated tonicity of smooth muscles in large and medium-sized conductive tubes constricts lumen diameter thereby compromising airflow and provoking asthma (Erle and Sheppard, 2014). In this view, a contraction-independent tension of the musculature may be needed for tracheal gas-filling in *Drosophila* embryos. Modulation of the balance between muscle tension and cuticle elasticity may, presumably, be responsible for adjusting the right decompression on the tracheal system at the end of embryogenesis when tracheal cuticle construction is completed ultimately promoting gas bubble formation, the last step in tracheal differentiation.

In summary, investigation of the cellular and molecular processes deployed during muscle-trachea communication in the *Drosophila* embryo may contribute to the research field of human pulmonic diseases including asthma.

## MATERIALS AND METHODS

### Fly husbandry and work

For embryo collection, flies were kept in cages on apple juice agar plates garnished with a spot of yeast at 25°C. Mutations (Table 2) were balanced over balancers that harbour a transgenic insertion of a *Dfd* promoter:*gfp* construct allowing unambiguous collection of homozygous mutant (non-green) embryos.

**Table 2.**
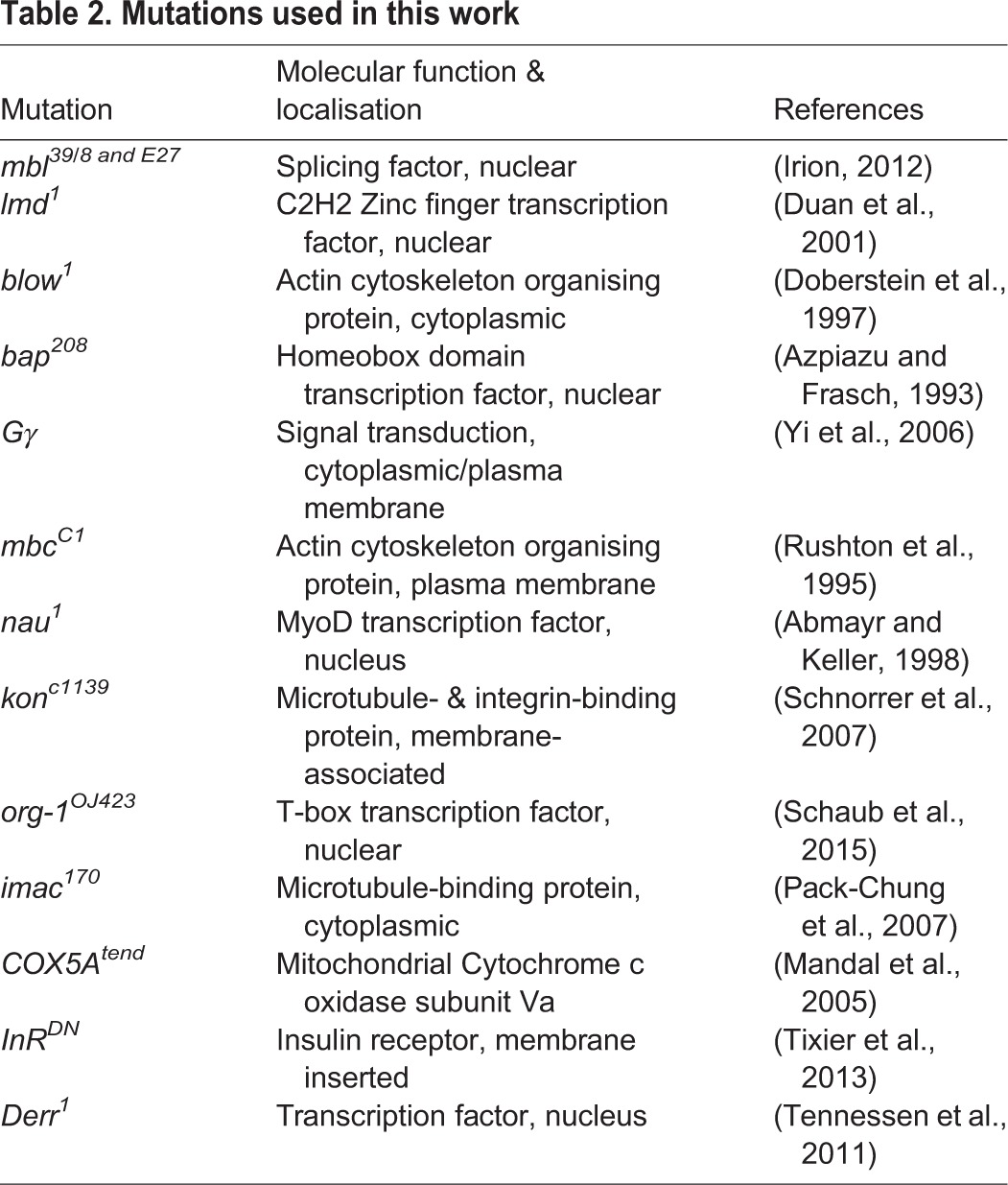
**Mutations used in this work**

For the rescue experiment shown in Table 1, we crossed flies that were heterozygous for *lmd* (on 3rd chromosome) and had a *UAS-lmd* insertion on the 2rd chromosome (the other 2rd chromosome being ‘wild-type’) to flies that were heterozygous for *lmd* and had a *twi-Gal4* insertion on the 2rd chromosome (the other 2rd chromosome being ‘wild-type’). Statistically, 25% of the progeny would be homozygous for the *lmd* mutation. Of these embryos 25% would receive both *UAS-lmd* and *twi-Gal4*. If there was a rescue, by consequence, 6.25% (25%×25%) of *lmd* homozygous embryos should have gas-filled tracheae. The expected value for non-rescued animals would then be 18.75%.

### Microscopy and imaging

To study gas-filling, embryos were collected at mid-stage 17 when the head skeleton and ventral denticles are not yet melanised. Wild-type embryos start to fill their tracheal system with gas after melanisation of these body parts. Embryos were observed on a Nikon AZ100 until their death (most mutants) or until they hatched using Nomarski optics. Pictures were taken from live embryos using the Nikon NIS software (Version 4.5). The rate of gas-filling is the percentage of embryos with gas-filled tracheae divided by the total number of embryos analysed.

For injection of the dyes Fluorescence Brightener 28 (Sigma Aldrich), 3 kDa and 10 kDa Dextran conjugated to rhodamine or FITC (Invitrogen), respectively, and the drugs ethanol and Philanthotoxin (Almone, 250 μM), embryos were dechorionated and mounted on a stripe of glue on a slide. Philanthotoxin was co-injected with 3 kDa FITC-conjugated dextran to verify successful injection. Eppendorf Femtojet was used for injection using manually produced glass needles. Philanthotoxin injected embryos were examined on a Zeiss microscope. Dye injected embryos were observed by a Zeiss 5Live line-confocal microscope. Usually, injected, and thereby wounded, embryos developed to hatching larvae, underlining that we were not analysing artifacts produced by dying animals. For transmission electron microscopy, we followed the protocol described by Moussian et al. (2006a). In brief, dechorionated embryos were immobilised by high-pressure freezing. They were fixed with glutaraldehyde by freeze substitution and embedded in Epon. Ultrathin sections were contrasted with lead citrate and uranyl acetate and viewed on a Philips CM10 electron microscope.
